# Review on the role of glutathione on oxidative stress and infertility

**DOI:** 10.5935/1518-0557.20180003

**Published:** 2018

**Authors:** Oyewopo Adeoye, Johnson Olawumi, Adeleke Opeyemi, Oyewopo Christiania

**Affiliations:** 1 Department of Anatomy, College of Health Sciences, University of Ilorin, Kwara State, Nigeria; 2 Department of Anatomy, College of Health Sciences, Osun State University, Osogbo, Nigeria; 3 Department of Anesthesia, University of Ilorin Teaching Hospital, Ilorin, Kwara State, Nigeria

**Keywords:** infertility, oxidative stress, glutathione, reactive oxygen species

## Abstract

Infertility is a global health problem and it is one of the most stressful conditions amongst married couples. Even though not lethal, it has been described as a radical life changing problem that carries with it significant psychological trauma. Infertility can be caused by various problems and sometimes it is not possible to establish a cause. Oxidative stress, which arises from an imbalance between reactive oxygen species (ROS) and protective antioxidants, influences the entire reproductive lifespan of men and women. ROS can modulate cellular functions, and oxidative stress can disturb the intracellular milieu, resulting in diseased cells or endanger cell survival. Under normal conditions, antioxidants act to oppose ROS production, scavenging existing free radicals and promoting the repair of ROS-induced damage to cell structures. At controlled levels, oxidative stress facilitates some physiological reproductive functions but at higher levels it is implicated in pathological processes in the reproductive tract that contribute to infertility and poor pregnancy outcomes. As high levels of reactive oxygen species and low antioxidant status have been implicated in conditions contributing to infertility, treatment based on strategies to boost the exhausted antioxidant defense of the reproductive microenvironment is intuitive. Glutathione is a natural body antioxidant, which helps preserve all other antioxidants. It is present in both the male and female gametes and its level varies widely. This study reviews the role oxidative stress plays in both male and female infertility, and the antioxidant action of glutathione on infertility.

## INTRODUCTION

Infertility is a problem of large magnitude, and is one of the most stressful conditions among married couples (Agarwal & Prabakaran, 2005). Even though it is not lethal, it has been described as a radical life changing problem that carries with it significant psychological trauma (Uadia & Emokpae, 2015). Infertility can be caused by different problems, and sometimes it is not possible to establish a cause. There may be a single cause in either partner or a combination of problems that may prevent conception occurring or a pregnancy continuing. Both men and women can have infertility problems, which is the case in about 20% of infertile couples. In around 15% of cases, no cause of infertility is identified in either partner and this is referred to as unexplained infertility. Combined female and male factor is responsible for 20-30% of cases. If the results of a standard infertility examination are normal, a diagnosis of unexplained or idiopathic infertility is assigned (Eskandari & Cadieux, 2003; Sekhon *et al*., 2010). However, when causes are identified among women, they most commonly include irregular ovulation, endometriosis and obstructed fallopian tubes; while among men, the most common cause are sperm disorders. Oxidative stress has a well-established role in the pathogenesis of explained and unexplained infertility, which is seen to affect 15% of couples (Eskandari & Cadieux, 2003; Sekhon *et al*., 2010).

Oxidative stress, which arises from an unbalance between reactive oxygen species (ROS) and protective antioxidants, influences the entire reproductive lifespan of men and women (Sekhon *et al.,* 2010). Reactive oxygen species may act as key signaling molecules in physiological processes, but in excess, uncontrolled levels may also mediate pathological processes involving the reproductive tract/reproduction. ROS can modulate cellular functions and oxidative stress (OS) can disturb the intracellular milieu, resulting in diseased cells or endanger cell survival. Under normal conditions, antioxidants act to oppose ROS production, scavenging existing free radicals and promoting repair of ROS -induced damage to cell structures (Agarwal & Allamaneni, 2004).

Glutathione is the mother of all antioxidants, the master detoxifier and maestro of the immune system (Hyman, 2011). It is one of the major endogenous antioxidant produced by cells participating directly in the neutralization of free radicals and reactive oxygen species, as well as maintaining exogenous antioxidants such as vitamins C and E in their reduced forms (Drigen, 2000).

This paper will review the role oxidative stress plays in both male and female infertility and the antioxidative action of glutathione on infertility and how the level of glutathione can be raised in the body.

### Oxidative Stress and Infertility

Infertility is the term used to describe a couple's failure to conceive, despite having engaged in regular and unprotected intercourse for a year (Robertson, 2015). It also includes the inability to carry a pregnancy to the delivery of a live baby (WHO, 1992). The World Health Organization (WHO, 1991) estimates that 8-12% of couples worldwide experience some forms of infertility during their reproductive lives, thus affecting 50-80 million couples, with 20-30 million in Africa. Therefore, it has been extrapolated that 3-4 million Nigerian couples are affected (Sule *et al.,* 2008). Even though infertility is not life threatening, it has been described as a radical life changing problem because it carries with it significant psychological trauma (Umezulike & Efetie, 2004). The prevalence of infertility in Sub-Saharan Africa ranges from 20- 40%. Although, the African socio-cultural setting has so far focused on females only, fertility problems are shared by both males and females (Uadia & Emokpae, 2015).

Oxidative stress (OS) affects the quality of gametes and the way in which they interact. Oxidative stress arises from an unbalance between pro-oxidant molecules generated from aerobic metabolism and protective antioxidants (Sekhon *et al.,* 2010). Free radicals such as ROS influence oocytes, spermatozoa and embryos and their environments. The microenvironments associated with follicular fluid, hydrosalpingeal fluid and peritoneal fluid have a direct bearing on oocyte quality, sperm oocyte interaction, sperm-mediated oocyte activation, implantation and early embryo development. OS affects early embryo development and implantation, which in turn affects pregnancy rates (Agarwal *et al.*, 2005a;b). Infertility is a problem of large magnitude and OS has been investigated as a causative factor.

### Oxidative stress and male infertility

Infertility probably affects at least one couple in six, and male factor infertility represents 30% of the diagnosis in infertile couples (Makker *et al.,* 2009). In Nigeria, the male factor is responsible for 40-50% of all infertility, although it varies from on region to region. The commonest singly defined cause of male factor infertility is sperm dysfunction (Hull *et al.,* 1985). High levels of ROS biomarkers were detected in semen samples from 25-40% of infertile men (Makker *et al.,* 2009). This may mean that ROS may be responsible, or it may be a contributory factor to the infertility been experienced by such individual; nevertheless, ROS have a physiological role in normal sperm function, acrosome reaction, hyperactivation, motility and capacitation of spermatozoa. Excessive levels of ROS may arise from immotile or morphologically abnormal spermatozoa and leukocytes (Sekhon *et al.,* 2010).

Spermatozoa are vulnerable to ROS because their plasma membrane and cytoplasm contain large amounts of polyunsaturated fatty acids (Alvarez & Storey, 1995), resulting in a decrease in sperm motility, presumably by a rapid loss of intracellular ATP, causing axonemal damage (de Lamirande & Gagnon, 1992), a decrease in sperm viability and an increase in mid-piece morphology defects, with deleterious effects on sperm capacitation and acrosome reaction. Lipid peroxidation of the sperm membrane is the key mechanism of this ROS-induced sperm damage, leading to infertility.

Excessive generation of ROS in the semen, by leukocytes as well as by abnormal spermatozoa, could be a cause of infertility (Sharma & Agarwal, 1996). Hydrogen peroxide is the major ROS producer in human spermatozoa. Moderately elevated concentrations of hydrogen peroxide do not affect sperm viability but cause sperm immobilization, mostly via depletion of intracellular ATP and the subsequent decrease in axonemal proteins' phosphorylation (Kemal Duru *et al.,* 2000; Misro *et al.,* 2004). High concentrations of hydrogen peroxide induce lipid peroxidation and results in cell death (Agarwal & Prabakaran, 2005).

A study reported that the levels of antioxidants in seminal plasma from infertile men were significantly lower than levels in fertile controls, and it was demonstrated that the levels of ROS produced by spermatozoa were negatively correlated with the quality of sperm in the original semen (Pasqualotto *et al.,* 2000). However, pathological levels of ROS detected in semen of infertile men are more likely a result of increased ROS production, rather than a reduced antioxidant capacity of the seminal plasma (Zini *et al.,* 1993). Virtually every human ejaculate is contaminated with potential sources of ROS such as leukocytes and abnormal spermatozoa. It follows that some spermatozoa will incur oxidative damage and a concomitant loss of function. Thus, the impact of ROS on male infertility is a question of degree rather than the presence or absence of the pathology.

### Oxidative stress and female infertility

Female infertility affects an estimated 48 million women with the highest prevalence affecting people in South Asia, Sub Saharan Africa, North Africa/Middle East, Central Europe and Central Asia (Mascarenhas *et al.,* 2012). Infertility affects women from around the world and the cultural and social stigma surrounding it varies. According to a study, the prevalence of female infertility ranges from 7% to 28% depending on the age of the woman (Yu & Yap, 2003). Although the frequency and origin of different forms of infertility varies, 40 - 50% of the etiology of infertility is due to female causes (Duckitt, 2003).

At controlled levels, free radicals can exert physiological effects and mediate processes such as tissue remodeling, hormone signaling, oocyte maturation, folliculogenesis, tubal function, ovarian steroidogenesis, cyclical endometrial changes, germ cell function, pregnancy, normal parturition and initiation of preterm labor (Agarwal *et al.*, 2005a;b). However, when ROS increase to pathological levels they are capable of inflicting significant damage to cell structures.

The pathological effects are exerted by various mechanisms including lipid damage, inhibition of protein synthesis and ATP depletion (Ray *et al.,* 2004). Oxidative stress plays a role in the etiopathogenesis of endometriosis, polycystic ovarian disease, hydatidiform mole, tubal factor infertility and unexplained infertility. There is growing literature on the effects of oxidative stress involved in the pathophysiology of pre-eclampsia (Tranquilli *et al.,* 2004), free induced birth defects (Loeken, 2004) and other situations such as abortions (łagód et al., 2001).

Oxidative stress induces infertility in woman through a variety of mechanisms. Excess ROS in the follicle may overwhelm follicular fluid antioxidant defense and directly damage oocytes. The DNA of oocytes and spermatozoa may be damaged, leading to defective fertilizations. Even when fertilization is achieved, oxidative stress-induced apoptosis may result in embryo fragmentation, implantation failure, abortion, impaired placentation and congenital abnormalities (Agarwal *et al.,* 2006). Excess reactive oxygen species may hinder the endometrium which normally functions to support the embryo and its development (Iborra *et al.,* 2005). Oxidative stress may induce luteal regression and insufficient luteal hormonal support for the continuation of a pregnancy (Agarwal & Allamaneni, 2004).

### The role of oxidative stress in some conditions that contribute to infertility

**Polycystic Ovarian Syndrome (PCOS):** PCOS is an anovulatory cause of infertility in 6-10% of premenopausal women (Asunción *et al.,* 2000). PCOS often can be characterized by hyper androgenism, hirsutism and oligomenorrhea or amenorrhea. Metabolic, endocrinological and cardiovascular disorders may coexist. Oxidative stress has been implicated in mediating the insulin resistance and increase in androgens seen in these patients (González *et al.,* 2006).

A recent study by Kuşçu & Var (2009) demonstrated increased MDA levels and upregulated SOD activity in patients' controls. MDA levels were highest in patients who exhibited insulin resistance. Insulin resistance and hyper glycemia are established as factors that increase oxidative stress. Fulghesu *et al.* (2002) evaluated the effect of N-acetyl-cysteine (NAC), known to replenish stores of the antioxidant glutathione, on insulin secretion and peripheral insulin resistance in subjects with PCOS.

**Endometriosis:** Endometriosis-associated infertility remains one of the most frustrating clinical situations encountered by the gynecologist. Although endometriosis is a common diagnosis in infertile couples, there remains a great deal of uncertainty about the mechanism and treatment of infertility in these patients. Severe cases of endometriosis are thought to render a woman infertile by mechanical hindrance of sperm-egg union by adhesions, endometriomas and pelvic anatomy malformations. ROS production may be amplified in the setting of endometriosis due to menstrual reflux, which subjects the peritoneal cavity to pro inflammatory hemoglobin and heme molecules released from transplanted erythrocyte debris (Reubinoff *et al.,* 1996). ROS are thought to promote the growth and adhesion of endometrial cells in the peritoneal cavity, contributing to the pelvic anatomical distortion known to cause infertility in endometriosis. Oxidative stress may have a role in promoting angiogenesis in ectopic endometrial implants by increasing vascular endothelial growth factor production (Park *et al.,* 2006).

Altered molecular genetic pathways may also contribute to the effects of oxidative stress in the pathogenesis of endometriosis and endometriosis-associated infertility. Differential gene expression of ectopic and normal endometrial tissue has been identified, including differential gene expression of glutathione-S-transferase, an enzyme in the metabolism of the potent antioxidant glutathione (Wu *et al.,* 2006). This suggests that altered molecular genetic pathways may determine the development of oxidative stress and its ability to induce cellular proliferation and angiogenesis in women with endometriosis.

**Unexplained Infertility:** The pathophysiology of unexplained infertility remains a scientific challenge. Elevated levels of ROS that disturb the redox balance within the body may be the root cause of infertility in women who do not have any other obvious cause. The ovum released from the ovary, the zygote or embryo and spermatozoa are vulnerable to damage inflicted by oxidative stress (Agarwal & Allamaneni, 2004). Wang *et al*. (1997) compared ROS levels in the peritoneal fluid between women undergoing laparoscopy for infertility evaluation and fertile women undergoing tubal ligation, and demonstrated that higher levels of ROS exist in the peritoneal fluid aspirated from patients with unexplained infertility, compared to that measured within the peritoneal fluid of fertile women. Polak *et al.* (1999) analyzed peritoneal fluid samples obtained at laparoscopy and found that women with unexplained infertility had increased MDA concentrations and TAS, implicating the role of redox unbalance in its pathogenesis. Elevated ROS levels in patients with unexplained infertility implies exhausted antioxidant defense, resulting in the inability to scavenge ROS and neutralize their toxic effects.

### Glutathione: The master antioxidant

Glutathione (GSH), tripeptide thiol is the major non-protein sulfhydryl compound in mammalian cells, known to have numerous biological functions. This thiol plays a prominent role in detoxification and antioxidation of exogeneous and endogenous compounds, as well as maintaining the intracellular redox status. It is a combination of three simple building blocks of protein or amino acids- cysteine, glycine and glutamine. Glutathione is a natural reservoir of reducing power, which can be quickly used by the cells as defense against oxidative stress. The sulfhydryl group (SH) of glutathione confers its protective action against oxidative damage. Glutathione exists in two forms: the reduced form (GSH) and the oxidized form (GSSG). The protective action of glutathione against reactive oxygen species (ROS) is facilitated by the interactions with its associated enzymes, such as glutathione peroxidase and glutathione reductase. In animal tissues, glutathione peroxidase, a selenium containing anti-oxidant enzyme, catalyzes the reduction of hydrogen peroxide and lipid peroxide in the presence of GSH, which is converted to GSSG. In turn, GSSG is reduced by glutathione reductase in the presence of nicotinamide adenine dinucleotide phosphate [NAD(P)H], which is generated mainly in the pentose phosphate pathway, as shown in the following equations:

$$\begin{array}{c} 2\mathrm{GSH}+H_{2}O_{2}\to \;\mathrm{GSSG}+2H_{2}O\\ \mathrm{GSSG}+\mathrm{NADPH}+H^{+}\to 2\mathrm{GSH}+{\mathrm{NADP}}^{+} \end{array}$$

Glutathione is a widely distributed thiol in animal organisms, not only in somatic cells but also in the gametes. In the body, the antioxidant defense capability consists of enzymatic and non-enzymatic systems, in which the latter is represented mainly by glutathione (Luberda, 2005). The body reproduces its own glutathione and it can be depleted by diet, pollution, toxins, medications, stress, trauma, aging, infections and radiation; and glutathione is normally recycled in the body.

The basic function of glutathione in the reproductive system is related to its interactions with other systems, as a preventive mechanism against ROS. Glutathione levels can be improved and optimized through the following ways: consuming sulphur-rich foods such as garlic, onions, cabbage, cauliflower, broccoli, etc., consuming bioactive whey protein found in non-pasteurized and non-industrially produced milk, which is a great source of cysteine and the amino acid building blocks for glutathione synthesis, doing exercise and taking a family of antioxidants which include vitamin C and E (in the form of mixed tocopherols), all working together to recycle glutathione (Nuttall *et al.,* 1998).

### Glutathione and male infertility

A glutathione deficiency can lead to instability of the sperm's mid piece resulting in defective motility (Hansen & Deguchi, 1996; Ursini *et al.,* 1999). It protects the plasma membrane from lipid peroxidation, scavenges superoxide and prevents oxygen formation. In a study consisting of infertile men with unilateral varicocele or genital tract inflammation, glutathione led to significant improvement in sperm quality (Lenzi *et al.,* 2004).

The glutathione/reductase system forms an excellent protection against the lipid peroxidation of the spermatozoa plasma membrane. It scavenges lipid peroxides, thereby arresting the progressive chain reaction of lipid peroxidation. It also scavenges hydrogen peroxide (H_2_O_2_), which is responsible for lipid peroxidation onset. Glutathione reductase stimulates the reduction of glutathione disulphide, to reduced glutathione, thereby recycling it.

### Glutathione and female infertility

Glutathione shields eggs from damage caused by oxidative stress during folliculogenesis, and as such, egg quality is dependent on it. In fact, research has shown that oocytes with higher levels of intracellular glutathione produce healthier and stronger embryos (Mukherjee *et al.,* 2014). Another study has shown that in younger years, women's ovaries have higher intracellular glutathione levels (Kankofer *et al.,* 2013).

It has been reported that glutathione deficiency is related to premature ovarian aging and even ovarian cancer (Lim *et al.,* 2013). Another study found that for women undergoing IVF, higher levels of glutathione in a woman's follicle translated into increased fertilization rates (Tola *et al.,* 2013). In other studies, glutathione is shown to be an antiaging antioxidant which could have possible impact on egg health, one of the cells most affected by the aging process (Fujii *et al.,* 2005). The protective action of follicle stimulating hormone on embryonic development is largely due to glutathione synthesis (Tsai-Turton & Luderer, 2006).

Glutathione can reduce oxidative stress by fighting the formation of damaging free radicals in the reproductive system (Gardiner *et al.,* 1998). It is the cell's primary antioxidant. Across the board, low levels of glutathione are a marker for disease and premature death. One of the areas of fertility, glutathione may have an impact on is the autoimmune issues. Glutathione is involved in regulating the genes that cause chronic inflammation. This may be helpful for those who are experiencing immunological miscarriages or if the body is rejecting one's mate's sperm.

## CONCLUSION

Oxidative stress occurs when the generation of reactive oxygen species (ROS) and other radical species overrides the scavenging capacity of antioxidants, either due to excessive ROS production or an inadequate availability of antioxidants. At controlled levels, oxidative stress facilitates some physiological reproductive functions but at higher levels it is implicated in pathological processes of the reproductive tract that contribute to infertility and poor pregnancy outcome. As high degrees of reactive oxygen species and low antioxidant status have been implicated in the conditions contributing to infertility, treatment based on strategies to boost the exhausted antioxidant defense of the reproductive microenvironment is intuitive.

Glutathione is the body's major antioxidant which helps in preserving all other antioxidants. It is a natural antioxidant present in both the male and female gametes, and its levels vary widely. It has been confirmed that it plays an important role in maintaining the biological value of germ cells, and it has been implicated in the fertilization process and early embryo development. The good thing is that the body can recycle glutathione if properly optimized, and it can also be destroyed.
